# Allied Ophthalmic Personnel: Backbone of Aravind’s Eye Care and Social Mission

**DOI:** 10.1038/s41433-026-04249-y

**Published:** 2026-01-21

**Authors:** Pranay Narang, Ravilla Ravindran, Thulasiraj Ravilla, Usha Kim, Veena Kannusamy, N. Venkatesh Prajna, Jeremy D. Keenan

**Affiliations:** 1 University of California, Berkeley/University of California, San Francisco Joint Medical Program, Berkeley, CA USA; 2 Aravind Eye Care System, Madurai, India; 3 Francis I. Proctor Foundation, University of California, San Francisco, CA USA; 4 Department of Ophthalmology, University of California, San Francisco, CA USA

**Keywords:** Education, Health services

As of 2020, an estimated 43 million people live with blindness globally – 80% of which is preventable or treatable [1] – and over 1.1 billion experience some degree of vision loss [2]. Nearly 25% of the world’s visually impaired population resides in India [3]. Addressing this challenge at scale demands care delivery models optimised for reach, efficiency, and equity. The Aravind Eye Care System (AECS), headquartered in Madurai, Tamil Nadu, has long strived toward these aims.

AECS was founded in 1976 as an 11-bed facility with the mission of eliminating needless blindness. AECS has since grown into one of the largest eye care providers globally, encompassing a network of hospitals, vision centres, community eye clinics, and eye banks. Each year, AECS performs more than 750,000 surgeries and serves 6.5 million patients [2, 4].

A key feature of its model is cross-subsidisation: half of all treatments and surgeries are provided at low or no cost for patients who cannot afford to pay. Hospitals are divided into “paying” and “free” sections, but surgical teams rotate across both, ensuring equivalent clinical quality. As of 2024, patients in the paying section may contribute ~15,000 INR (180 USD) for cataract surgery, while those in the free section may pay ~1000 INR (12 USD). Outreach camps further extend AECS’s reach into remote areas, enabling early screening and access to care [5].

AECS has been the subject of prominent case studies for its relevance to other health systems [6]. While the average U.S. ophthalmic surgeon performs ~350 cataract operations per year, AECS surgeons average around 1500, often conducting six to eight cataract surgeries hourly. Furthermore, complication rates remain low, reportedly less than half those at UK hospitals [7]. Although other institutions in India also deliver eye care at scale, AECS is known for combining surgical volume, cost-efficiency, and equitable access [8].

While AECS’s operational efficiency has been documented, one foundational component remains underexamined: the Allied Ophthalmic Personnel (AOP) program. This program not only enables AECS’s surgical volume but also employs thousands of rural women facing limited economic opportunity, facilitating upward social mobility. Despite its centrality to AECS – and implications for gender equity, care delivery, and workforce innovation – the AOP has not been characterised. This commentary highlights how AECS is addressing workforce shortages while uplifting a vulnerable population to meet the rising demand for ocular care.

The demand for eye care in India is immense, yet ophthalmologists and nursing personnel are scarce and costly. Accordingly, AECS developed the AOP model for delegation of clinical, administrative, and perioperative responsibilities that need not be performed by an ophthalmologist, such as intraocular pressure measurement, refraction, and perioperative preparation. Today, this model is key in enabling surgeons to operate in a continuous assembly-line” [9–12].

In designing the AOP, AECS leadership considered the socioeconomic context of South India, where young women in rural areas face poverty, exploitation, and disproportionately limited opportunities. In place of the conventional nursing personnel, this population became the focus of recruitment for the AOP program to train rural women – 17-18 years olds with no prior work experience.

AECS identified that this population, having only completed higher secondary education, could be trained not only in clinical skills but also in the institution’s core values. They undergo two years of comprehensive, residential training covering anatomy, ocular diseases, pharmacology, microbiology, and clinical skills. Upon graduation, they are equipped to use various ophthalmic instruments for tasks such as assessing visual acuity, measuring intraocular pressure, and performing lacrimal syringing. They triage and support ophthalmologists in managing ocular emergencies such as chemical burns, corneal injuries, acute glaucoma, and foreign bodies. They assist surgeons in the operating room, maintaining instrument counts and preparing patients in rapid succession [13]. AECS also prioritises cultivating traits such as honesty, integrity, punctuality, and teamwork, embedding a value-driven ethos that defines the culture across the institution. Referred to as “sisters” and identified by their white uniforms, AOPs are regarded as the backbone of AECS.

After recruitment, AOP receive two years of free training during which their living and food accommodations are provided free of cost. The social and economic support during the training period is crucial for these young girls to continue in the program. Upon graduation, which requires completing a written exam and interview, AOP are hired with a monthly salary of over 21,000 INR as of 2025. In this role, AOP take on responsibility for daily hospital operations, exercising decision-making authority that keeps services running and affirms their empowerment. Their salary increases, with continued living and free accommodations. The AOP typically work for four years, often leaving for marriage. Notably, “sisters” are encouraged to save a sizable portion of their salary, which accumulates into ~600,000 INR as of 2024 over this period. These savings help these young women to marry well with the ability to cover wedding costs, including dowry payments which are still prevalent in the region.

The AECS actively supports AOP as they transition into marriage, encouraging unions where women are treated with respect. In cases where former AOPs have experienced domestic violence, they have been welcomed back to AECS with indefinite employment, accommodation, and progressively increasing salary. Some of these women have gone on to purchase homes, raise children independently, and assume academic roles within the institution, including conducting and publishing research. Others who marry have secured positions in private practices, where they earn higher compensation.

The impacts of this pathway for rural women are multifold: providing training and employment following high school; offering job security and financial stability, both within AECS and private practice; supporting the ability to marry and raise a family; and ensuring safety and protection – a central reason women choose to enrol.

Through the AOP, AECS has created a model of workforce innovation and social empowerment that sustains one of the world’s highest-volume surgical care systems. By employing historically marginalised women, the program offers insight into how health systems can be designed not only to deliver care, but to uplift local communities and advance equity.
